# Supplementary material for: Cultivar Discrimination of Single Alfalfa (Medicago sativa L.) Seed via Multispectral Imaging Combined with Multivariate Analysis
Source: Sensors (Basel). 2020 Nov 18;20(22):6575. doi: 10.3390/s20226575 (PMC7698633; doi:10.3390/s20226575)
Supplement: Supplementary file 1 [file sensors-20-06575-s001.pdf]

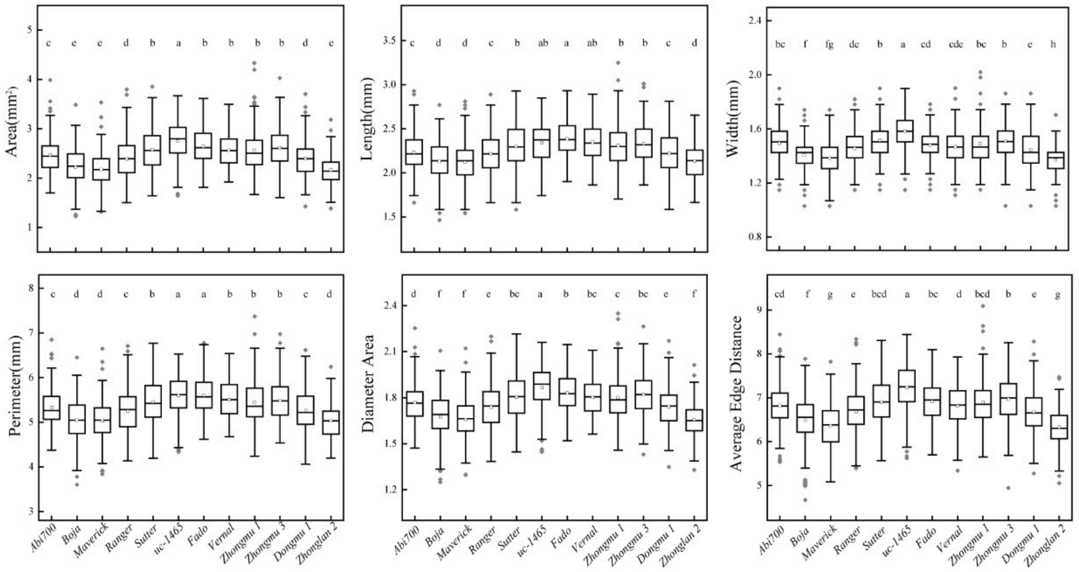

Supplementary Figure S1. The binary features of 12 *Medicago sativa* L. cultivars. Different letters indicate significant differences among cultivars at the same binary feature.

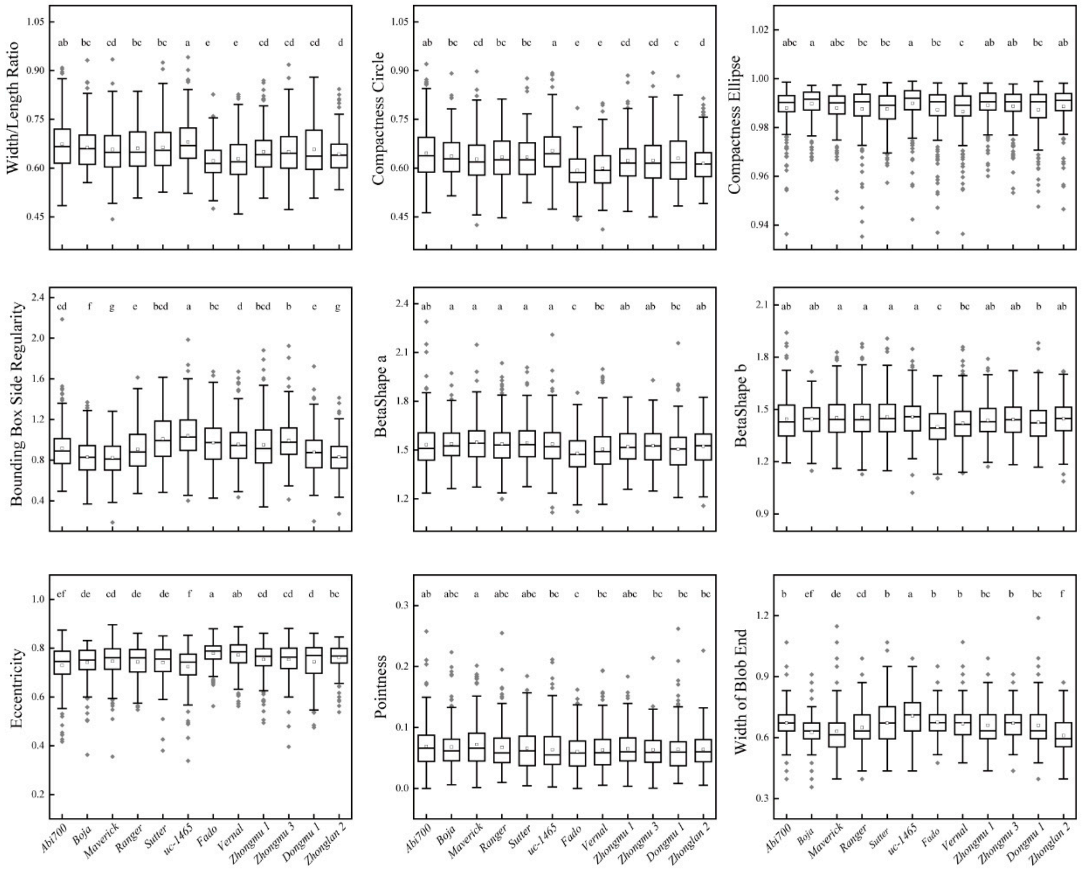

Supplementary Figure S2. The shape features of 12 *Medicago sativa* L. cultivars. Different letters indicate significant differences among cultivars at the same shape feature.

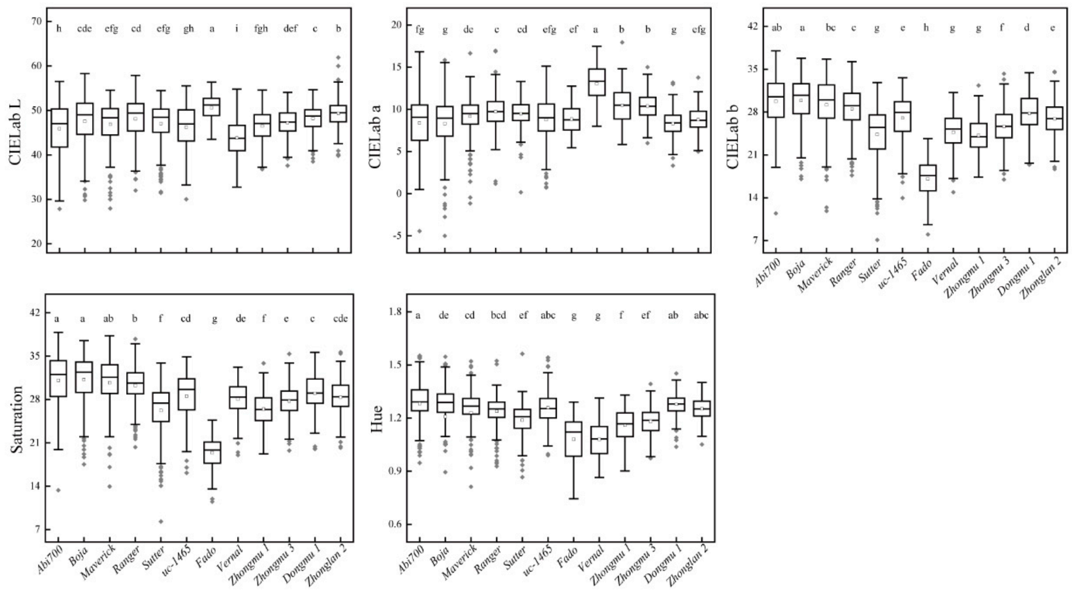

Supplementary Figure S3. The color features in 12 *Medicago sativa* L. cultivars.

Different letters indicate significant differences among varieties at the same color feature.

Supplement Table S1. List of the extracted variables from multi spectral images.

Name	Feature	Description
Area(mm^2)	Binary Feature	Area of blob.
Length(mm)	Binary Feature	Length of blob bounding box.
Width(mm)	Binary Feature	Width of blob bounding box.
Perimeter	Binary Feature	Returns length of blob perimeter.
Diameter Area	Binary Feature	Diameter of a circle with the same area as the blob.
Average Edge Distance	Binary Feature	Returns average distance to the edge from a pixel inside the blob.
Width/Length Ratio	Shape Feature	Ratio of width to length of the image oriented bounding box.
Compactness Circle	Shape Feature	Compactness of blob defined as 4*Area/(π*length^2), ratio of object area to the area of a circle with the same length (isolength quotient).
Compactness Ellipse	Shape Feature	Compactness of blob defined as 4*Area/(π*length*width), ratio of object area to the area of an ellipse with the same length and width (isolength quotient).
Bounding Box Side Regularity	Shape Feature	Regularity of the four sides of a blob, when oriented to the smallest enclosing rectangle within a selection of blob angles. Regularity measure include stdDev and range for the following sequence.
BetaShape a	Shape Feature	Parameter a of beta-ellipse fitted to blob mask. Parameter a corresponds to width of most pointed blob-end
BetaShape b	Shape Feature	Parameter b of beta-ellipse fitted to blob mask. Parameter b corresponds to width of least pointed blob-end
Eccentricity	Shape Feature	Sqrt (Length^2 - Width^2)/Length.
Pointness	Shape Feature	Pointness of the blob defined as: (2* Half largest area - Total area)/ Total area.
Width of Blob End	Shape Feature	Returns width of blob at either widest or narrowest end.
CIELab L*	Color Feature	Mean Luminance component of CIELab-color of blob.
CIELab a*	Color Feature	Mean A-component of CIELab-color of blob.
CIELab b*	Color Feature	Mean B-component of CIELab-color of blob.
Saturation	Color Feature	Mean saturation of blob based on CIELab coordinates according to formulae: S = SQRT (A^2 + B^2).
Hue	Color Feature	Mean hue of blob based on CIELab coordinates according to formulae: H = ATAN (B/A).

Supplementary Table S2. Mean reflectance of each cultivar at different spectral wavelength.

Wavelength	Variety										
	Abi700	Boja	Maverick	Ranger	Sutter	uc-1465	Fado	Vernal	Zhongmu1	Zhongmu3	Dongmu1	Zhonglan2
365	6.53h	6.62h	6.71h	7.09g	7.53f	6.60h	12.21a	7.75e	8.93b	8.64c	8.22d	9.01b
405	6.89g	7.04fg	7.25f	7.58e	8.26d	7.03fg	12.32a	7.78e	9.22b	8.84c	8.24d	9.17b
430	7.43h	7.70f	7.77g	8.22f	8.87d	7.59gh	12.56a	8.21f	9.68b	9.30c	8.63e	9.64b
450	9.12g	9.70f	9.45f	10.09e	10.43d	9.45f	13.72a	9.50f	10.96c	10.85c	10.23de	11.22b
470	10.31hi	10.96f	10.66g	11.28e	11.40e	10.57gh	14.30a	10.10i	11.70cd	11.76c	11.46de	12.40b
490	11.21g	11.89de	11.61ef	12.14cd	12.14cd	11.40fg	14.72a	10.60h	12.26c	12.44c	12.42c	13.27b
515	12.78f	13.50de	13.18ef	13.64d	13.35de	12.83f	15.58a	11.69g	13.33de	13.63d	14.05c	14.69b
540	13.00g	13.75de	13.46ef	13.88de	13.59e	13.07fg	15.88a	12.01h	13.74de	14.04cd	14.37c	15.05b
570	16.06c	16.70b	15.97c	16.98b	15.82c	15.55c	17.65a	14.37d	15.93c	16.04c	16.96b	17.57a
590	17.35ef	18.13cd	17.63def	18.54bc	17.45ef	17.10f	19.13a	16.17g	17.42ef	17.75de	18.47bc	19.02ab
630	18.95g	19.87cde	19.78def	20.52bc	19.68def	19.15fg	21.45a	19.24efg	19.70def	20.24cd	20.34bcd	20.97ab
645	19.51f	20.45de	20.65cd	21.28bc	20.65cd	19.89ef	22.34a	20.44de	20.67cd	21.28bc	21.00cd	21.76ab
660	19.57f	20.60de	21.04cd	21.60bc	21.26cd	20.00ef	22.85a	21.29cd	21.21cd	21.82bc	21.19cd	22.19ab
690	22.54e	23.61cd	24.15bc	24.53b	24.17bc	23.21de	25.84a	24.66b	24.09bc	24.70b	24.09bc	24.84b
780	32.63bc	33.13ab	32.60bc	32.60bc	31.59d	33.56a	33.37a	33.51a	31.76d	32.05cd	32.08cd	31.44d
850	42.28bcd	42.75ab	42.35bc	42.41bc	41.44e	43.25a	42.74ab	42.99a	41.74de	41.91cde	41.54e	40.66f
880	45.88bcd	46.41ab	46.04bc	46.12b	45.16ef	46.84a	46.20b	46.34ab	45.42def	45.56cde	45.02f	44.06g
940	53.77c	54.53ab	54.41b	54.28b	53.57cd	54.92a	53.61cd	53.45cd	53.17d	53.31cd	52.43e	51.00f
970	56.04c	56.88ab	56.84b	56.63b	56.04c	57.26a	55.66cd	55.40d	55.29d	55.41d	54.48e	52.82f

Different letters indicate significant differences among cultivars at the same wavelength.

Supplementary Table 3. Discrimination performance based on LDA with morphology features of 12 *Medicago sativa* L. cultivars.

	Actual	Predict												
		Abi700	Boja	Maverick	Ranger	Sutter	uc-1465	Fado	Vernal	Zhongmu1	Zhongmu3	Dongmu1	Zhonglan2	Total (%)
Training (n=140)	Abi700	80	26	23	10	1	14	0	2	0	2	10	3	
	Boja	14	44	13	9	1	2	0	0	2	1	9	6	
	Maverick	11	23	47	19	2	2	0	3	3	1	8	6	
	Ranger	10	23	25	31	5	11	0	6	3	12	8	6	
	Sutter	2	0	3	3	50	7	0	6	25	19	13	13	
	uc-1465	11	3	4	12	14	72	0	4	8	16	14	1	
	Fado	0	0	0	0	1	0	140	1	3	0	0	1	
	Vernal	4	5	7	13	3	5	0	84	28	19	2	1	
	Zhongmu1	0	0	1	3	18	2	0	11	38	24	6	3	
	Zhongmu3	1	3	3	11	12	7	0	16	11	21	4	3	
	Dongmu1	5	4	4	17	14	15	0	2	6	12	47	18	
	Zhonglan2	2	9	10	12	19	3	0	5	13	13	19	79	
	Accuracy (%)	57.14	31.43	33.57	22.14	35.71	51.43	100.00	60.00	27.14	15.00	33.57	56.43	43.63
Testing (n=60)	Abi700	30	11	8	4	0	9	0	1	0	0	6	0	
	Boja	10	18	6	1	1	1	0	0	1	0	3	2	
	Maverick	3	9	17	10	0	0	0	4	1	0	4	2	
	Ranger	4	10	13	16	1	4	0	1	3	4	2	0	
	Sutter	1	0	1	1	20	2	0	3	10	13	5	5	
	uc-1465	5	2	0	5	3	32	0	1	2	8	6	1	
	Fado	0	0	0	0	0	0	60	0	2	0	0	0	
	Vernal	3	3	6	4	3	0	0	37	7	7	1	0	
	Zhongmu1	1	0	0	2	9	0	0	6	18	9	2	4	
	Zhongmu3	0	0	0	4	4	4	0	7	5	7	3	2	
	Dongmu1	2	3	3	8	7	5	0	0	3	6	14	11	
	Zhonglan2	1	4	6	5	12	3	0	0	8	6	14	33	
	Accuracy (%)	50.00	30.00	28.33	26.67	33.33	53.33	100.00	61.67	30.00	11.67	23.33	55.00	41.94

Supplementary Table 4. Discrimination performance based on LDA with spectral features of 12 *Medicago sativa* L. cultivars.

		Predict												
	Actual	Abi700	Boja	Maverick	Ranger	Sutter	uc-1465	Fado	Vernal	Zhong mu1	Zhong mu3	Dongm u1	Zhongl an2	Total (%)
Training (n=140)	Abi700	113	2	0	20	0	0	0	0	0	0	0	0	
	Boja	10	107	1	21	0	1	0	0	0	0	0	0	
	Maverick	0	0	114	0	9	11	0	0	0	0	0	0	
	Ranger	17	29	0	99	0	0	0	0	0	0	0	0	
	Sutter	0	0	7	0	127	8	0	0	0	0	0	0	
	uc-1465	0	2	18	0	4	120	0	0	0	0	0	0	
	Fado	0	0	0	0	0	0	140	1	0	0	1	0	
	Vernal	0	0	0	0	0	0	0	126	0	5	2	0	
	Zhongmu1	0	0	0	0	0	0	0	5	127	2	11	0	
	Zhongmu3	0	0	0	0	0	0	0	5	0	133	2	0	
	Dongmu1	0	0	0	0	0	0	0	3	13	0	124	0	
	Zhonglan2	0	0	0	0	0	0	0	0	0	0	0	140	
	Accuracy (%)	80.71	76.43	81.43	70.71	90.71	85.71	100.00	90.00	90.71	95.00	88.57	100.00	87.50
Testing (n=60)	Abi700	48	2	0	10	0	0	0	0	0	0	0	0	
	Boja	4	47	0	13	0	0	0	0	0	0	0	0	
	Maverick	0	0	50	0	1	4	0	0	0	0	0	0	
	Ranger	8	10	0	37	0	0	0	0	1	0	0	0	
	Sutter	0	0	2	0	59	4	0	0	0	0	0	0	
	uc-1465	0	1	8	0	0	52	0	0	0	0	0	0	
	Fado	0	0	0	0	0	0	60	1	0	0	0	0	
	Vernal	0	0	0	0	0	0	0	54	0	0	2	0	
	Zhongmu1	0	0	0	0	0	0	0	2	49	1	8	0	
	Zhongmu3	0	0	0	0	0	0	0	1	0	59	0	0	
	Dongmu1	0	0	0	0	0	0	0	2	10	0	50	0	
	Zhonglan2	0	0	0	0	0	0	0	0	0	0	0	60	
	Accuracy (%)	80.00	78.33	83.33	61.67	98.33	86.67	100.00	90.00	81.67	98.33	83.33	100.00	86.81

Supplementary Table 5. Discrimination performance based on LDA with morphology and spectral features of 12 *Medicago sativa* L. cultivars.

		Predict												
	Actual	Abi700	Boja	Maverick	Ranger	Sutter	uc-1465	Fado	Vernal	Zhongmu1	Zhongmu3	Dongmu1	Zhonglan2	Total (%)
Training (n=140)	Abi700	119	3	0	11	0	0	0	0	0	0	0	0	
	Boja	8	125	1	7	0	0	0	0	0	0	0	0	
	Maverick	0	1	132	0	2	14	0	0	0	0	0	0	
	Ranger	13	11	0	121	0	0	0	0	0	0	0	0	
	Sutter	0	0	3	1	134	2	0	0	0	1	0	0	
	uc-1465	0	0	4	0	4	124	0	0	0	0	0	0	
	Fado	0	0	0	0	0	0	140	0	0	0	0	0	
	Vernal	0	0	0	0	0	0	0	129	0	3	0	0	
	Zhongmu1	0	0	0	0	0	0	0	1	125	1	9	0	
	Zhongmu3	0	0	0	0	0	0	0	5	0	134	1	0	
	Dongmu1	0	0	0	0	0	0	0	5	15	1	130	0	
	Zhonglan2	0	0	0	0	0	0	0	0	0	0	0	140	
	Accuracy (%)	85.00	89.29	94.29	86.43	95.71	88.57	100.00	92.14	89.29	95.71	92.86	100.00	92.44
Testing (n=60)	Abi700	48	0	0	5	0	0	0	0	0	0	0	0	
	Boja	3	53	0	4	0	0	0	0	0	0	0	0	
	Maverick	0	0	58	0	0	8	0	0	0	0	0	0	
	Ranger	9	7	0	50	0	0	0	0	0	0	0	0	
	Sutter	0	0	2	1	59	1	0	0	0	0	0	0	
	uc-1465	0	0	0	0	1	51	0	0	0	0	0	0	
	Fado	0	0	0	0	0	0	60	0	0	0	0	0	
	Vernal	0	0	0	0	0	0	0	56	0	2	0	0	
	Zhongmu1	0	0	0	0	0	0	0	0	52	1	4	0	
	Zhongmu3	0	0	0	0	0	0	0	4	0	57	1	0	
	Dongmu1	0	0	0	0	0	0	0	0	8	0	55	0	
	Zhonglan2	0	0	0	0	0	0	0	0	0	0	0	60	
	Accuracy (%)	80.00	88.33	96.67	83.33	98.33	85.00	100.00	93.33	86.67	95.00	91.67	100.00	91.53
